# Single Actin Bundle Rheology

**DOI:** 10.3390/molecules22101804

**Published:** 2017-10-24

**Authors:** Dan Strehle, Paul Mollenkopf, Martin Glaser, Tom Golde, Carsten Schuldt, Josef A. Käs, Jörg Schnauß

**Affiliations:** 1 Faculty of Physics and Earth Sciences, Peter Debye Institute, Leipzig University, Linnéstr. 5, 04103 Leipzig, Germany; dan.strehle@gmx.net (D.S.); paul.mollenkopf@uni-leipzig.de (P.M.); martin.glaser@uni-leipzig.de (M.G.); tom.golde@uni-leipzig.de (T.G.); schuldt@physik.uni-leipzig.de (C.S.); jkaes@physik.uni-leipzig.de (J.A.K.); 2 Fraunhofer Institute for Cell Therapy and Immunology (IZI), DNA Nanodevices Group, Perlickstraße 1, 04103 Leipzig, Germany

**Keywords:** biopolymers, actin, bundles, optical tweezers, rheology, mechanical properties, dynamics

## Abstract

Bundled actin structures play an essential role in the mechanical response of the actin cytoskeleton in eukaryotic cells. Although responsible for crucial cellular processes, they are rarely investigated in comparison to single filaments and isotropic networks. Presenting a highly anisotropic structure, the determination of the mechanical properties of individual bundles was previously achieved through passive approaches observing bending deformations induced by thermal fluctuations. We present a new method to determine the bending stiffness of individual bundles, by measuring the decay of an actively induced oscillation. This approach allows us to systematically test anisotropic, bundled structures. Our experiments revealed that thin, depletion force-induced bundles behave as semiflexible polymers and obey the theoretical predictions determined by the wormlike chain model. Thickening an individual bundle by merging it with other bundles enabled us to study effects that are solely based on the number of involved filaments. These thicker bundles showed a frequency-dependent bending stiffness, a behavior that is inconsistent with the predictions of the wormlike chain model. We attribute this effect to internal processes and give a possible explanation with regard to the wormlike bundle theory.

## 1. Introduction

The cytoskeleton is a meshwork of a variety of biopolymers, providing eukaryotic cells with mechanical stability and dynamic functions, which affect a cell’s structure and function [1,2]. A dominating cytoskeletal component is the semiflexible polymer actin, which, for instance, builds up the actin cortex and is involved in crucial cellular processes such as wound healing, embryonic development, tissue engineering, and immune response [1,3,4,5,6,7,8].

Being one of the most relevant polymers in terms of mechanical impact on cell properties, actin has been subject to numerous studies, both theoretical and experimental [4,9,10,11,12,13,14,15,16,17,18]. Remarkably, individual filaments can be arranged in different structures such as networks or bundles and their interplay with crosslinking molecules enables a rich phase space of mechanical responses against external stimuli. This vast phase space is further enriched by the transience of these crosslinkers such as fascin, fibrin, and α-actinin, which bind and unbind with rates in the order of tenths to tens of seconds [19,20,21]. These varying structural isoforms are ubiquitous in the cytoskeleton, rendering it a composition of diversified structures [11]. To decouple the effects of the differing structures, it is essential to study them in a bottom-up, isolated fashion. Following this approach, single actin filaments and networks have been investigated rigorously with respect to their mechanical properties [4,11,12,13,14,15,16,17,18]. The mechanics of bundled structures and the molecular design principles responsible for their mechanical properties, however, still remain poorly understood. A major drawback is that bundles are highly anisotropic impeding studies employing established conventional approaches, such as bulk rheology, that rely on isotropic networks. The mechanical characterization of bundles relies on elaborate experiments on the µm-scale to determine their properties. Pioneering examples for these investigations are the determination of bundles’ bending stiffness measured by observing passive contour undulations induced by thermal fluctuations [21], and the evaluation of time-dependent mechanical responses of crosslinked actin bundles after deformations actively induced via optical tweezers [20,22]. These experimental studies are accompanied by new theoretical approaches within the so-called worm-like bundle model aiming to develop an understanding of the microscopic picture of bundle mechanics. However, a complete rheological characterization has not been feasible so far, since main characteristics such as frequency-dependent mechanics could not be tested.

**Figure 1:**
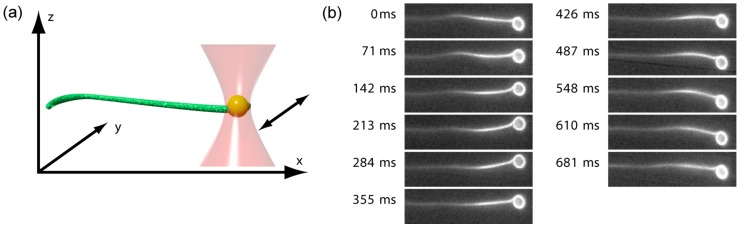
(**a**) A 2-μm polystyrene bead coated with streptavidin is attached to an actin bundle enriched with biotinylated actin monomers. This bead is trapped by optical tweezers, and an oscillatory movement of the trap in the *xy*-plane induces oscillations in the bundle. The oscillation amplitudes subsequently decay when traveling through the bundle, a process which can be captured by fluorescence microscopy; (**b**) Fluorescence images of the experimental procedure. The 11 images represent half a period at a frequency of 0.7 Hz.

Here, we resolve this methodological limitation and present a novel approach based on optical tweezers technology. Optical tweezers were used to actively deform actin bundles to rheologically characterize these structures and their responses for different frequencies. The concept is based on the method previously described by Riveline et al. [23], and the theoretical framework introduced by Wiggins et al. [24] that describes elastic rods under the influence of hydrodynamic drag, thereby allowing the deduction of the filaments’ stiffness from its contour shape. We adapted and extended this approach to actin filaments bundled by depletion forces, using methyl cellulose as a depletion agent. Compared to the work by Riveline et al., we measured bundles with larger spatial extensions and observed a total decay of the induced oscillation, as illustrated in Figure 1. We found that thin bundles obey the theoretical predictions of the worm-like chain model [25]. To investigate the impact of bundle thickness, we introduced an approach to thicken bundles by merging them in a controlled manner (see Materials and Methods). These thickened bundles exhibited a differing mechanical behavior, in particular, they showed a frequency-dependent stiffness, which is reminiscent of viscoelastic materials. Treating bundling as an unspecific type of crosslinking, this result may be explained by the consensus of the wormlike bundle (WLB) theory [15,26]. As our system does not contain specific crosslinking proteins, which have been assumed by the theoretic model, this result is not inherent with respect to the corresponding theory. We suppose that previously reported mechanisms such as velocity-dependent inter-polymer sliding friction [27] and bundle relaxation [7] play a significant and non-negligible role, which is not yet completely understood. Transitions between systems dominated by crosslinking and systems which are determined by friction and relaxation may be rather indistinct.

## 2. Results

**Figure 2:**
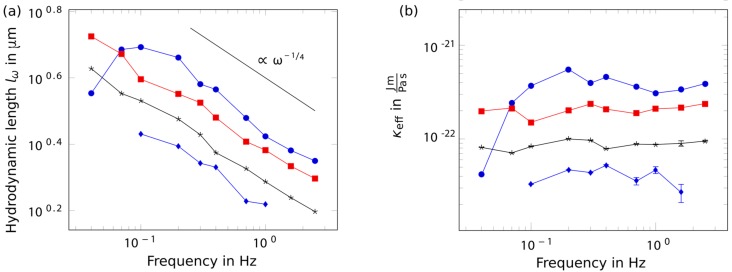
Hydrodynamic length and persistence length. Different colors and markers depict different individual bundles of varying thicknesses (blue dotted: thin bundle, blue circles: thick bundle, others: intermediate). (**a**) Regarding the frequency dependence of the hydrodynamic length, wiggling bundles with a bead attached to their ends revealed a power law exponent of −1/4; (**b**) which translated into a constant bending stiffness. Few bundles, however, also showed a softening behavior towards low frequencies.

After an appropriate constellation of a bundle attached to a bead was found and adjusted with respect to its angle to the oscillation direction, the system was exposed to excitations spanning a frequency range between 0.04 Hz and 2.5 Hz. The experimental performance was limited by the sensitivity of the camera and the sample’s degradation caused by bleaching effects. The minimum exposure time of 50 ms delimitated the temporal resolution for high frequencies (50 ms extended over one fifth of one period at 4 Hz). On the other side, 0.04 Hz already corresponded to an oscillation period of 25 s. As several periods must be recorded to minimize transient effects, photo-bleaching of the sample was a crucial limitation. As a first test, we verified that bundles did not change their behavior with time by exposing bundles to a defined and fixed driving frequency over a time interval of 600 s. We observed no overall tendency to diverge from an average value for the measured hydrodynamic length (data not shown). For the frequency tests, bundles were excited by a standard set of driving amplitudes with predefined frequencies, a method that had been introduced by Riveline et al. to study single filaments [23]. The unique combination with the evaluation processing, based on the theoretical framework by Wiggins et al. [24], allowed us to rheologically study bundle structures for the first time. The acquired hydrodynamic length shows a scaling with frequency lω∝ω−14, as predicted by the theory for a wormlike chain (WLC) (see Figure 2a) translating to a constant bending rigidity $\kappa_{eff}=\frac{\kappa }{\zeta }=l_{\omega }^{4}\cdot \omega \;$ (see Figure 2b). The drag coefficient ζ is a function of the Reynolds number and scales with the ratio Ld, where L denotes the contour length and d the bundle diameter. In our experiments this ratio was only subject to minimal variations since the length of the bundles exceeded their according diameters by orders of magnitudes (L >> d). Consequently, the impact of thickness variations (Δd) to the drag coefficient are negligible. The amplitude decay of tested bundles yielded a bending stiffness in the order of $10^{-21}\frac{J\;m}{Pa\cdot s}$. For comparison, for a bundle of 50 tightly crosslinked filaments measured in a medium with a viscosity of η=50 mPa·s, a bending rigidity of $1.5\times 10^{-19}\frac{J\;m}{Pa\cdot s}$ was observed [28]. While most of the bundles obeyed the prediction of the WLC model and displayed a stiffness invariance over the whole frequency range, it was conspicuous that rather thick bundles (see Figure 2b: blue dotted) showed a softening behavior for low frequencies. This indicated stress relaxation mechanisms, which are not accounted for by the wormlike chain model.

**Figure 3:**
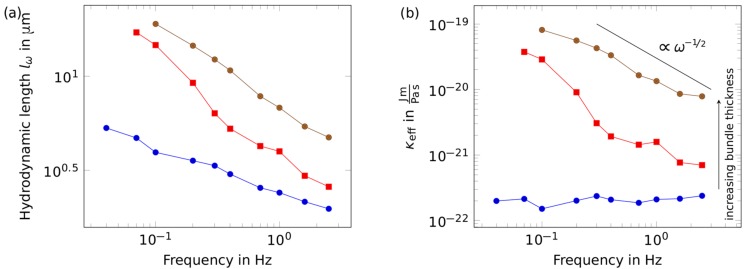
Rheological response of a successively thickened bundle. (**a**) While comparably thin bundles (blue circles) obey the predictions determined by the wormlike chain model, thicker bundles (intermediate: red squares, thick: brown circles) deviated from the ω−14-scaling, which translates into a frequency-dependent bending stiffness (**b**). With more and more bundles merged, the bending stiffness increased. At the same time, the bundles stiffened markedly for lower frequencies.

The thickness of an individual bundle cannot be determined sufficiently, as emphasized previously [20]. A theoretically possible approach to extrapolate the bundle thickness from thermal fluctuation [29] by observation of the attached microbead is impeded by the fact that bundle twist and bending contributions cannot be separated in an exact manner. A further constraint is photo-bleaching effects, rendering experiment times too short to gain an adequate dataset for statistically significant values. Comparing the fluorescence intensities of bundles to those of single filaments at least enabled a rough estimation of the number of filaments in a bundle. This approach yielded bundle sizes of 20 to 70 filaments per bundle. Using the technique to merge bundles described above, the thickness of one specific bundle was increased successively between measurements, verifying that indeed a thinner and a thicker bundle, i.e., different numbers of filaments, are compared. The bundle stiffness increased with increasing thickness (see Figure 3a). However, in contrast to the measurements on single bundles with lower thicknesses, the bending stiffness—instead of being constant—showed a scaling with the frequency according to κ∝ω−12. The bending stiffness gradually increases for lower frequencies, which is in contrast to some of the a priori thick bundles that showed softening behavior for low frequencies. Inherently, different frequencies agitated bundles on different length scales, which were governed by the hydrodynamic length. Thus, thickening a bundle led to a stiffening, and probing on an extended length scale resulting in a higher bending stiffness for longer bending modes (see Figure 3b).

## 3. Discussion

Within this manuscript, we introduce a novel method to study highly anisotropic bundled filament structures and investigate the frequency dependence of their mechanic response. By monitoring the propagation of an oscillatory motion excited by amplitudes induced by an optical tweezers setup, the bending stiffness of the bundle was determined for varying oscillation frequencies between 0.04 Hz and 2.5 Hz. The hydrodynamic length was derived from the decay of the oscillation amplitude, which was proven to be constant for long time measurements over 600 s, excluding mechanically exited degradation effects and bundle instabilities. Bundles were formed and stabilized by depletion forces, allowing us to study the mechanical effects exclusively caused by the filaments and their inter-filament interactions. We would like to note that bundles cannot be treated as homogenous rods since the contour of the bundle highly depends on the involved filaments, which are not equally long but rather reveal a length distribution, leading to a slightly varying diameter along the entire structure. These indeterminable uncertainties surely influence the experiments and may result in small deviations in measured hydrodynamic lengths. However, we consider these deviations to be negligible. In preliminary experiments, we made sure that defects, kinks, or homogeneities were measurable only if distinguishable under the microscope. Mechanical responses of filament bundles exposed to wiggling excitations varied in magnitude, but the majority of the tested bundles obeyed the predictions derived from the WLC model. The scaling of the hydrodynamic length with the frequency revealed a power law exponent of −14. With $\kappa_{eff}\propto l_{\omega }^{4}$, this translates into a constant effective bending stiffness over the whole frequency range, which was also assumed by the WLC model. Consequentially, these bundles can be described as semiflexible wormlike chains with bending stiffness as their defining material property. 

However, a number of bundles showed a softening behavior at very low frequencies. This was conspicuous at frequencies corresponding to oscillation periods larger than 5 s. These observations coincide with the theory for viscoelastic materials, which release the strain-induced stress over time. As a consequence, these bundles appeared stiffer when exposed to high frequencies, since the stress relaxation becomes increasingly dominant at distinctively larger time scales [4,30,31]. Conversely, they showed a softening behavior at low frequencies, when stresses can be relaxed due to a restructuring of the material, a mechanism which was also indicated by earlier experiments [20]. Actively bending actin bundles and holding them in a deformed state for a sufficient time facilitated a reconstruction of the material, illustrating an internal relaxation [20]. At low frequencies and large time scales, individual filaments within a bundle are able to slide against each other. This is captured in the theoretical framework provided by the worm like bundle theory, which explains the softening of a bundle with a decreasing contribution of crosslinking to the bending stiffness [15,32,33]. In the spirit of this theory, bundle cohesiveness is less pronounced under these circumstances.

Composite bundles were formed by iteratively merging individual bundles simply by converging two of them, a process we denoted as the zipping effect. Although we were not able to produce bundles of defined thicknesses (since the measurement of this quantity was not feasible for the aforementioned reasons), this method allowed us to compare the mechanical properties of one particular bundle with increasing thickness. The mechanical responses of these thickened bundles were remarkably different from the responses of simple a priori thick bundles. For comparably thin bundles, the scaling of the hydrodynamic length with frequency was the same as that for individual bundles and followed the relation lω∝ω−14. However, a significant increase in thickness yielded a change of the entire mechanical appearance. These bundles displayed a stiffness increase towards lower frequencies, which is in stark contrast to the previous rheological measurements in this study, which showed a softening of bundles probed for low frequencies.

**Figure 4:**
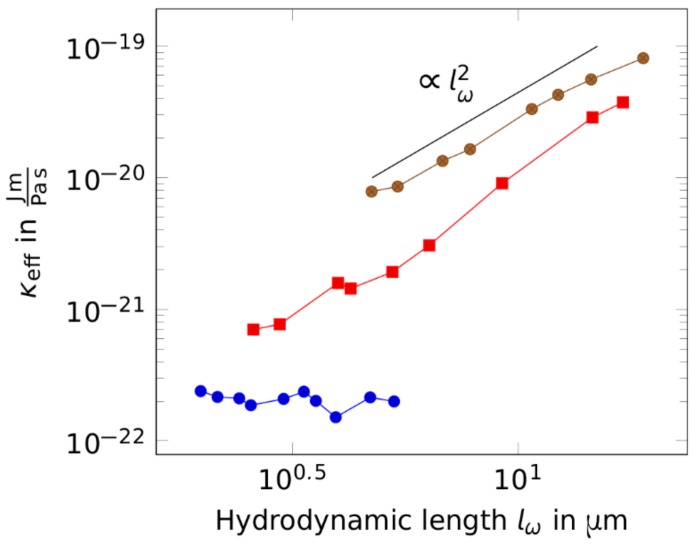
Stiffness scaling with the hydrodynamic length for increasing bundle diameters (thin bundles: blue circles, intermediate bundles: red squares, thick bundles: brown circles). For thickened bundles, the bending stiffness shows a scaling behavior with the excitation frequency. This scaling is more evident when looking at the bending stiffness with respect to the hydrodynamic length. While probing the bundle with different frequencies, the length scale on which the bundle was deformed changed. This translates into a higher bending stiffness for longer deformation modes.

This counterintuitive behavior becomes more evident when having a closer look at the expression of the hydrodynamic length, which sets the length scale over which the bending of the driven bundle decays. The length of the bundles’ deformation mode is determined by the hydrodynamic length. Hence, probing bundles at lower frequencies is equivalent to measurements on longer lengths, leading to the interpretation that thick bundles appear stiffer at longer lengths (see Figure 4). A similar result has been reported by Taute et al. [34] as well as Pampaloni et al. [35], who observed a length-dependent stiffness of microtubules. Microtubules serve as a model system for wormlike bundles due to their protofilament architecture [15,26]. Composed by multiple protofilaments laterally connected by highly reversible tubulin-tubulin bonds, they react elastically to small deformations while undergoing continuous deformation characteristics for long wavelength modes [36]. For contour lengths L>5 μm, fluctuation measurements revealed a relaxation time scaling $\tau \propto L^{4}$, as predicted by the wormlike chain model for semiflexible polymers [34]. For shorter contour lengths, the relaxation time scaled with the microtubule length as $\tau \propto L^{2}$. Measured persistence lengths significantly increased with filament length, but exhibited a plateau value for filaments smaller than 5 μm, which was also predicted by the wormlike bundle theory, proposing that different stiffness scalings are inherently connected with different regimes [15,26]. For actin bundles induced by the depletion agent polyethylene glycol (PEG), a behavior was observed that has been associated with a regime describing highly coupled bundles [37]. Interestingly, we observed a scaling behavior that fits the predictions made by the WLB theory for a regime where the bundle response to bending is dominated by the shearing of crosslinkers. This is rather surprising, since we formed bundles exclusively by depletion forces without any crosslinking proteins. However, another recent study on bundles that also did not involve specific crosslinking proteins revealed that molecular crowding and electrostatic interactions lead to an elastic coupling between filaments [22]. The stiffness of the relevant bending modes was predicted to scale with the wave vector in the form of $\kappa_{n}\propto q_{n}^{-2}$, according to the WLB theory. Crosslinker shearing as well as filament bending and stretching were assumed to contribute approximately equally to the bending energy of the bundle [15]. Since, in our experiments, one mode was predominantly excited due to wiggling at the fixation point with a wave length of $q^{-1}\propto l_{\omega }$, the effective bending stiffness for thick bundles was not constant, but rather scaled with the hydrodynamic length as $\kappa_{eff}\propto l_{\omega }^{2}$ and with the frequency as κeff∝ω−12. The consensus of these predictions shows that mechanisms contributing to the mechanical appearance of anisotropic bundled structures are versatile and still not completely understood. One of these contributions might be inter-filament friction. Ward et al. [27] recently reported unexpectedly large inter-polymeric forces due to friction in bundled systems scaling logarithmically with the sliding velocity. Additionally, Schnauß et al. [7,38] found contractile forces acting against the lateral extraction of single filaments out of a bundle, which facilitates an exponential relaxation after stresses (e.g., caused by motion in a viscous medium) are released. It is very likely that both friction and bundle relaxation influence the frequency-dependent response of bundles. These types of interactions in a fully coupled regime are not yet covered by the WLB model. The phenomenological distinction between these contributions and the comparison to crosslinked systems is not trivial; however, all of these processes are based on inter-filament interactions, which can be captured in the frame of stick-slip models [39].

Further measurements on depletion force-induced bundles, as well as on bundles crosslinked by specific proteins, are necessary to separate these diverse contributions to the mechanical response of a bundle. The method presented here is well-suited since it provides the possibility to actively excite filamentous bundles and thereby covers the investigation of frequency-dependent frictional effects. The comparison of depletion force-induced bundles to bundles crosslinked by α-actinin should isolate the influences of crosslinker shearing and friction in combination with bundle relaxation, respectively.

In conclusion, we introduce a new and powerful tool providing remarkable insights into the rheological nature of filamentous bundles. Our initial data revealed perceptions of frequency-dependent mechanical responses of such bundles, which have not been experimentally accessible before.

## 4. Materials and Methods

### 4.1. Protein Preparation, Bead Functionalization, and Bundle Formation

G-actin was prepared from rabbit skeletal muscle as described previously by Smith et al. [40] according to the protocols established by Humphrey et al. [41]. Monomeric actin was polymerized at a concentration of 5 µM in F-buffer (0.1 M KCl, 1 mM MgCl_2_, 0.1 mM CaCl_2_, 0.5 mM TrisHCl, pH 7.8) in the presence of phalloidin-Tetramethylrhodamine B isothiocyanate (TRITC) (Sigma-Aldrich, St. Louis, MO, USA) overnight [42]. Biotinylated G-actin (Cytoskeleton, Inc., Denver, CO, USA) was added to one tenth of the actin concentration and subsequently incubated at room temperature. Biotin-actin was thereby incorporated at the ends of the already-formed filaments. 

Polystyrene beads, 2 µm in diameter and functionalized by a covalently bound streptavidin coat (Polysciences, Inc., Warrington, PA, USA), were added in an additional incubation period. Streptavidin and its counterpart biotin form a highly selective ligand-receptor bond with a very low dissociation constant. This facilitates the attachment of the functionalized beads predominantly to the ends of the filaments enriched with the accumulation of biotinylated actin. 

The composition of the final solution in F-buffer was diluted to a total actin concentration of 200 nM in the presence of polystyrene beads and glucose/glucose oxidase as an anti-bleaching agent. The F-buffer contained 0.8% methyl cellulose (400 or 4000 cP, Sigma-Aldrich, St. Louis, MO, USA) acting as a bundling agent by inducing depletion forces.

About 10 μL of the final solution was deposited on a Sigmacote-treated (Sigma-Aldrich, St. Louis, MO, USA) squared coverslip with a 22-mm edge length and vacuum grease-lined edges. A 24 mm × 50 mm coverslip was placed on top of the drop and then pressed flat, with care taken to remove trapped air pockets. The sample chamber was pressed together between two aluminum plates in a controlled fashion with a micrometer screw, as previously described by Strehle et al. [20]. The 2-µm beads were free in solution and trappable in the potential of a focused laser beam pertaining to an optical tweezers setup. In the best-case scenario, a bead was already attached to an actin bundle; however, this attachment could have been achieved by approaching the bead to the end of a free bundle in the solution.

### 4.2. Optical Tweezers Setup and Fluorescence Microscopy

For the presented experiments, an optical tweezers setup was used, as previously described in [7]. Sample observation and simultaneous recording of fluorescence microscopy images during experimental procedures were performed with a Hamamatsu Orca ER digital CCD (charge-coupled device) camera (Hamamatsu Photonics Deutschland GmbH, Herrsching am Ammersee, Germany).

For data acquisition, all components were controlled and integrated by a self-written LabView (National Instruments, Munich, Germany) program that allowed visualization of fluorescent beads and rhodamine-phalloidin labeled F-actin [42]. Fluorescence images of beads and bundles were recorded, and bead positions were determined in evaluation by cross-correlation analysis.

### 4.3. Experimental Procedure

Bundling F-actin can be achieved by different approaches. One possibility is to introduce additional crosslinking proteins such as α-actinin in a sufficiently high concentration to facilitate a parallel alignment of filaments to increase the number of available binding sites [8,43]. Here, the investigated anisotropic filament structures were depletion force-induced bundles in order to avoid additional interactions between the components. 

**Figure 5:**
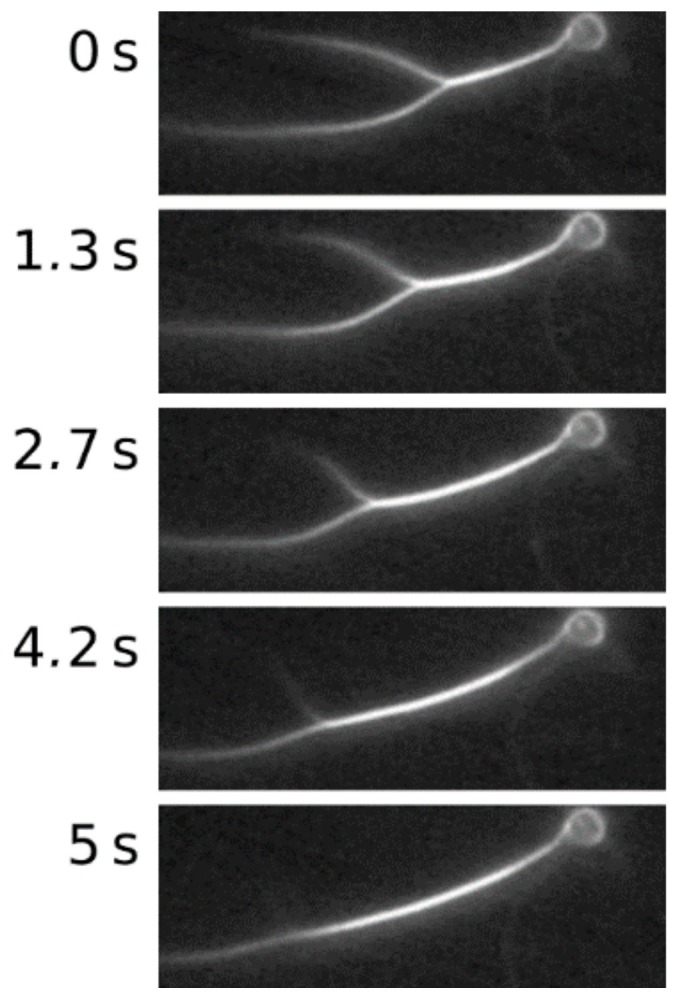
Time lapse of the zipping process. Bundles were thickened by merging with other bundles. This was achieved by dragging the bundle with the optically trapped bead into the vicinity of another, freely floating bundle.

In the presence of non-interacting polymers, an attractive force is generated between colloidal filaments. In the spirit of the model by Asakura and Osaka [44], polymers are treated as freely interpenetrating hard spheres excluded from the colloid surface by a thin layer. This shell creates a positive free energy difference, which is lowered if two colloidal particles share this excluded volume. Consequently, the total entropy of the system is increased and thus the free energy of the system is decreased [45]. These bundles were found to be able to merge easily when approaching each other solely due to the described entropic effect. Exploiting this behavior, a rather simple method to attain increasingly thick bundles was conceived. This “zipping”- process (see Figure 5) was used to increase the bundle thickness successively between measurements.

Bundles were mechanically excited by moving the attached polystyrene beads via optical tweezers with defined amplitudes in the *y*-direction at varying frequencies. The position of the optical trap was controlled via a self-written LabView software enabling the wiggling procedure in a controlled and reproducible fashion (see Figure 1). Phalloidin-TRITC-labeled filaments were observed with fluorescence microscopy and recorded with a CCD camera, which allowed us to trace the bundle contours over time. 

Obtained images were processed for evaluation in several steps. First, the image was preset geometrically for a better handling in evaluation. The respective direction of deflection was rotated and set as the *y*-direction, while the bundle contour in the undisturbed state was rotated and set as the *x*-direction. Subsequently, the largest anticipated amplitude was chosen as the boundary and the image was cropped to the corresponding width. Afterwards, a filter was applied, marginally smoothing the images in the *x*-direction averaging with a Gaussian profile while also enhancing edges (emphasizing horizontal lines) in the *y*-direction. This enabled the application of a parabola fit to the contour and, by the determination of the parabola’s maximum, the bundle deflection was defined.

### 4.4. Analytical Tools

Elastic rods under the influence of hydrodynamic drag [23] were described theoretically by Wiggins et al. [24] by solving a differential equation for the motion of a filament under deformation by hydrodynamic flow, which was derived from the Hamiltonian for a wormlike chain. In the weakly-bending rod limit and for small deformations, the bending force for a WLC can be written as:

$$f_{bend}=-\kappa y_{xxxx}{\hat{e}}_{y},$$

where κ denotes the bending stiffness and subscripts the partial derivatives with respect to the subscripted variable. The experiments took place at low Reynolds numbers, where the bending force is balanced by the hydrodynamic drag, which is represented by a velocity-dependent force:

$$f_{drag}=\zeta (y_{t}-u){\hat{e}}_{y},$$

with background velocity u and friction coefficient ζ. This gives the equation of motion:

$$\zeta (y_{t}-u)=-\kappa y_{xxxx}.$$

Rescaling the distance along the filament with the hydrodynamic length scale, we get:

lω=(κωζ)14=(kBTlpωζ)14,

which sets the scale on which the filaments’ displacement amplitude decays. Using a product ansatz for appropriate border conditions gives the solution for a filament driven at one end as

$$y\left(\eta ,t\right)=\frac{y_{0}}{2}\left[e^{-\tilde{S}\eta }\cos\left(\tilde{C}\eta -\omega t\right)+e^{-\tilde{C}\eta }\cos\left(\tilde{S}\eta +\omega t\right)\right].$$

with $\eta =\frac{x}{l_{\omega }},\;\tilde{C}≃0.92$ and $\tilde{S}≃0.38$ this solution describes two waves decaying on different time scales; the excitation wave in the first term and its reflection by the medium in the second term. A similar ansatz for a filament driven at its middle point yields the solution for one side of the contour:

$$y\left(\eta ,t\right)=\frac{y_{0}}{2}\left\{e^{-\tilde{S}\eta }\left(\cos\left(\tilde{C}\eta -\omega t\right)+\sin\left(\tilde{C}\eta -\omega t\right)\right)+e^{-\tilde{C}\eta }\left(\cos\left(\tilde{S}\eta +\omega t\right)+\sin\left(\tilde{S}\eta +\omega t\right)\right)\right\}.$$

**Figure 6:**
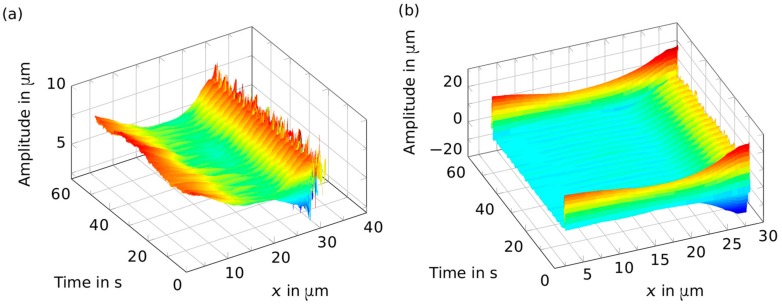
Wiggling bundle position data. Panel (**a**) shows the amplitude of the movement of the bundle contour in the *y*-direction with respect to its point along the bundle. The bead that is controllably deflected by the optical tweezers setup to induce oscillations is near position *x* = 27 µm, and is attached to one end of the bundle. Bundles that are not orthogonal to the oscillation direction were excluded from further analysis. Panel (**b**) shows the data after band passing. The first and last periods need to be excluded from $l_{\omega }$ -fitting because of the border effects of filtering

Figure 6a illustrates the recorded bundle positions over time. Eminent spikes are a direct result of noisy bundle recognition, especially in the bead vicinity. Non-orthogonal bundles, i.e., bundles that were not aligned orthogonal to the driving direction for their entire length, were excluded from further analysis since they did not show substantial oscillations. To eliminate random contributions due to thermal undulations as well as poor bundle detections, a band pass filter was used to isolate the oscillatory bundle movement with the driving frequency.
